# J. Rolin Root

**Published:** 1842-03

**Authors:** 


*Obituary.*—Died in Hamilton village, on Tuesday morning, 30th ult. after a short illness, of gastro-enteritis, J. ROLIN ROOT, Dentist, aged 30 years.

Mr. Root had been but between one and two years a citizen of this place; but in that short time, by his peaceful and friendly temper, his strict integrity, his suavity of manners, and his faithfulness and skill in his profession, he had rapidly succeeded in securing the good will, friendship and confidence of the community. His untimely death will be deeply deplored as a public loss, by a numerous circle of acquaintances and friends. He has left a wife and an infant child.—*Palladium.*

